# Magnetic Resonance Imaging Evaluation of the Accuracy of Various Lymph Node Staging Criteria in Rectal Cancer: A Systematic Review and Meta-Analysis

**DOI:** 10.3389/fonc.2021.709070

**Published:** 2021-07-13

**Authors:** Zixuan Zhuang, Yang Zhang, Mingtian Wei, Xuyang Yang, Ziqiang Wang

**Affiliations:** Department of Gastrointestinal Surgery, West China Hospital, Sichuan University, Chengdu, China

**Keywords:** rectal cancer, magnetic resonance imaging, metastasis, lymph node, lymph node staging, node-by-node

## Abstract

**Background:**

Magnetic resonance imaging (MRI)-based lymph node staging remains a significant challenge in the treatment of rectal cancer. Pretreatment evaluation of lymph node metastasis guides the formulation of treatment plans. This systematic review aimed to evaluate the diagnostic performance of MRI in lymph node staging using various morphological criteria.

**Methods:**

A systematic search of the EMBASE, Medline, and Cochrane databases was performed. Original articles published between 2000 and January 2021 that used MRI for lymph node staging in rectal cancer were eligible. The included studies were assessed using the QUADAS-2 tool. A bivariate random-effects model was used to conduct a meta-analysis of diagnostic test accuracy.

**Results:**

Thirty-seven studies were eligible for this meta-analysis. The pooled sensitivity, specificity, and diagnostic odds ratio of preoperative MRI for the lymph node stage were 0.73 (95% confidence interval [CI], 0.68–0.77), 0.74 (95% CI, 0.68–0.80), and 7.85 (95% CI, 5.78–10.66), respectively. Criteria for positive mesorectal lymph node metastasis included (A) a short-axis diameter of 5 mm, (B) morphological standard, including an irregular border and mixed-signal intensity within the lymph node, (C) a short-axis diameter of 5 mm with the morphological standard, (D) a short-axis diameter of 8 mm with the morphological standard, and (E) a short-axis diameter of 10 mm with the morphological standard. The pooled sensitivity/specificity for these criteria were 75%/64%, 81%/67%, 74%/79%, 72%/66%, and 62%/91%, respectively. There was no significant difference among the criteria in sensitivity/specificity. The area under the receiver operating characteristic (ROC) curve values of the fitted summary ROC indicated a diagnostic accuracy rate of 0.75–0.81.

**Conclusion:**

MRI scans have minimal accuracy as a reference index for pretreatment staging of various lymph node staging criteria in rectal cancer. Multiple types of evidence should be used in clinical decision-making.

## Introduction

Rectal cancer has become the leading cause of cancer-related deaths in China and worldwide. By 2030, it is estimated that there will be approximately 2.2 million cases (1, 2). The determination of lymph node staging remains a significant challenge in rectal cancer treatment. Lymph nodes at a risk of metastasis in rectal cancer are mainly located in the mesentery and usually range in size from 1 to 10 mm. Lymph node status is the most important determinant of local recurrence and overall survival (3).

According to the National Comprehensive Cancer Network (NCCN) and American Joint Committee on Cancer (AJCC) staging standards (4, 5), lymph node invasion should be evaluated before treatment to guide the formulation of treatment plans. Patients with lymph node involvement can benefit from preoperative neoadjuvant therapy, considerably reducing the local recurrence rate. However, over-treatment of the lymph node stage may lead to genitourinary system damage and other consequences (6, 7). Therefore, accurate preoperative staging is essential for providing patients with the optimal treatment.

The diagnostic methods currently used for preoperative lymph node staging include magnetic resonance imaging (MRI), computed tomography (CT), and endoscopic ultrasound (EUS). MRI can accurately display the mesorectal fascia, the depth of tumor invasion, circumferential resection margin (CRM), and extramural venous invasion (EMVI), and it has now become the gold standard for preoperative staging and re-staging in local areas (8).

Unfortunately, the results of previous studies have shown that MRI has a poor performance in detecting metastatic lymph nodes (9, 10). At present, there are various diagnostic criteria for metastatic lymph nodes, including size, shape, and boundaries, that have been widely discussed. However, there is no consensus on the accurate diagnosis of metastatic lymph nodes (11–13).

Four previous meta-analyses assessed the accuracy of MRI for lymph node staging of rectal cancer but did not differentiate the lymph nodes defined by different morphological standards (14–17). Additionally, the included studies only used histological results to assess the lymph node status indirectly and did not directly assess lymph nodes on MRI scans. The studies did not perform a histological examination of each lymph node in the specimen so that the position of each lymph node was accurately matched with its corresponding MRI scan, allowing for the node-by-node comparison of MRI scans and histological results to accurately analyze the status of each lymph node.

To the best of our knowledge, this study is the first systematic review and meta-analysis of the accuracy of various lymph node staging criteria in rectal cancer with MRI and includes the literature that contained the node-by-node correspondence between MRI scans and histopathologic results for analysis. To more accurately evaluate the accuracy of MRI in the pretreatment staging of rectal cancer lymph nodes, we hope to obtain more detailed results by synthesizing a large number of published studies.

## Methods

### Search Strategy

A comprehensive search of Medline (January 2000–January 2021), Embase (January 2000–January 2021), and the Cochrane Database (2000–January 2021) was performed according to the Preferred Reporting Items for Systematic Reviews and Meta-Analyses (PRISMA) (18) by two investigators (ZZX and ZY), using index terms “((((((((N-stage) OR (Nodal staging)) OR (Lymph node)) OR (Diagnostic imaging)) OR (mesorectal lymph nodes)) OR (Neoplasm Staging)) OR (Lymphatic Metastasis))) AND (((“Magnetic Resonance Imaging”[Mesh]) AND (“Rectal Neoplasms”[Mesh])) AND (sensitiv*[Title/Abstract] OR sensitivity and specificity[Mesh Terms] OR (predictive[Title/Abstract] AND value*[Title/Abstract]) OR predictive value of tests[Mesh Term] OR accuracy*[Title/Abstract])) as text words. The last search was on January 10, 2021.

### Study Selection

Studies were included based on the following criteria: 1) original articles on the diagnostic performance of MRI in the staging of rectal cancer, 2) a phased-array MRI coil was used for imaging, 3) histopathologic findings were used as reference standards, 4) the reference criteria for assessing metastatic lymph nodes were clearly mentioned, and 5) sufficient data were available to calculate true-positive, false-positive, false-negative, and true-negative values.

The exclusion criteria were as follows: 1) inclusion of patients with non-rectal cancer, 2) research using other less common MRI types, 3) assessment of staging according to a non-Tumor–Node–Metastasis (TNM) staging system, 4) inclusion of patients who received preoperative chemoradiotherapy, 5) articles that were not original research articles, such as reviews, letters, or case reports, 6) repeated publications.

Titles and abstracts identified by the search strategy were independently reviewed by two reviewers. For all abstracts that met the inclusion criteria or were potentially eligible, full articles were retrieved and independently reviewed by two reviewers. Disagreements were resolved by consensus or by discussion with a third reviewer. All included studies followed the PICOS criteria.

### Data Extraction and Quality Assessment

Two reviewers independently extracted the data. The following data was collected: (year of publication, sample size, country), study design (prospective or retrospective), MRI protocol (field strength and resolution parameters), reference criteria for assessing metastatic lymph nodes, and blinding procedure.

The diagnostic results were calculated on a lesion level for each outcome: Patients/lymph nodes with histologically confirmed lymph node metastasis are classified as node-positive (pN+), regardless of the number of metastatic lymph nodes. Patients/lymph nodes without any metastatic lymph nodes are classified as node-negative (pN-).

The QUADAS-2 evaluation tool was used to evaluate the quality of all studies in the systematic review.

### Statistical Analysis

Meta-analysis and the associated I^2^ statistic were evaluated with Meta-Disc 1.4(Ramón y Cajal Hospital, Madrid, Spain) and Stata 16.0(STATA Corporation, College Station, TX, USA) (19).

The threshold effect was evaluated using Spearman’s correlation coefficient of the logit of sensitivity and logit of 1-specificity.

A bivariate random-effects model was used to summarize diagnostic statistics and displayed using summary receiver operating characteristics (SROC) plots.

Meta-regression and subgroup analyses were performed to detect heterogeneity. Additionally, a sensitivity analysis was conducted (20).

Publication bias was evaluated with an asymmetry test and a Deek’s funnel plot assessment using Stata 16.0 (21).

## Results

### Description of Included Studies

**Figure 1:**
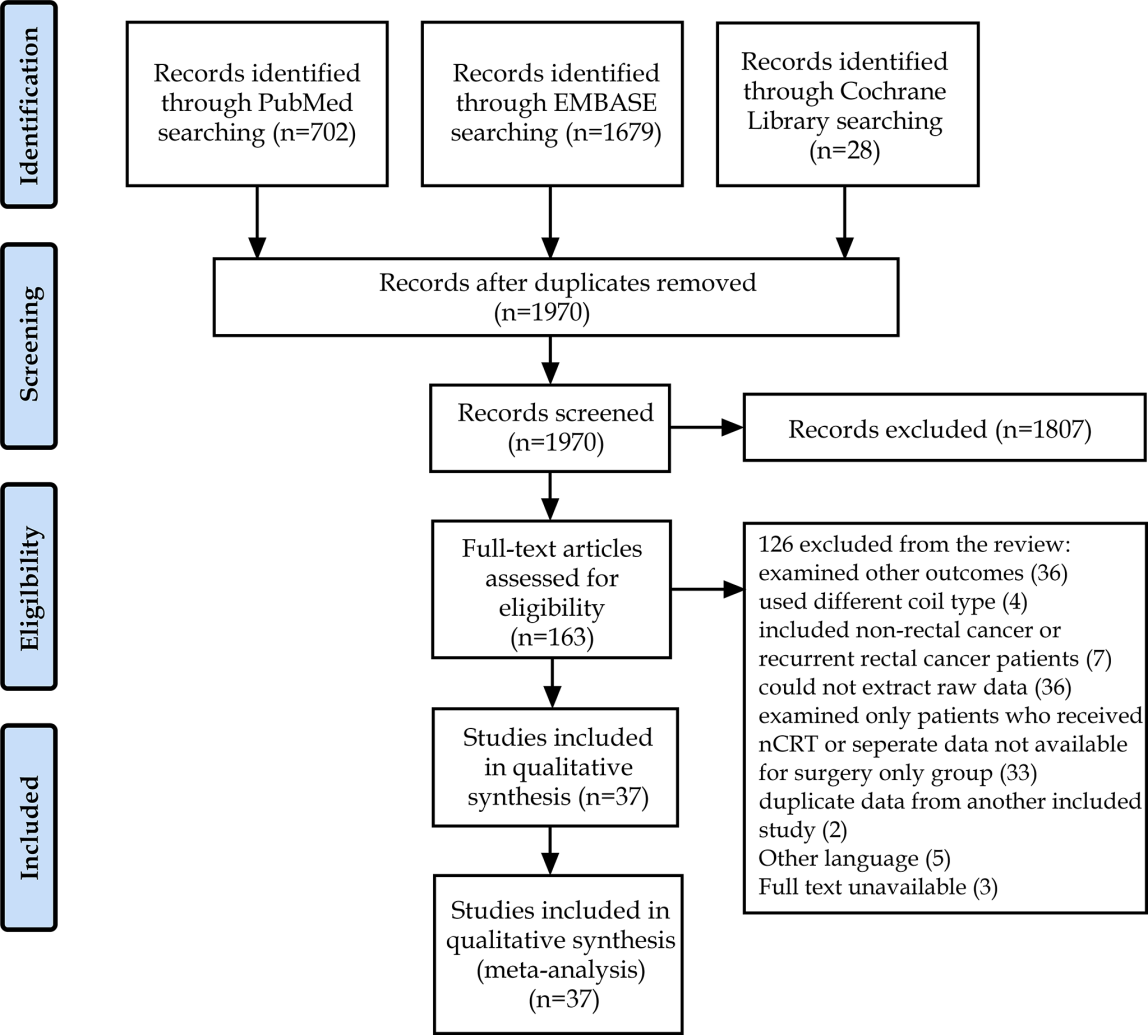
Flow chart according to PRISMA.

**Figure 2:**
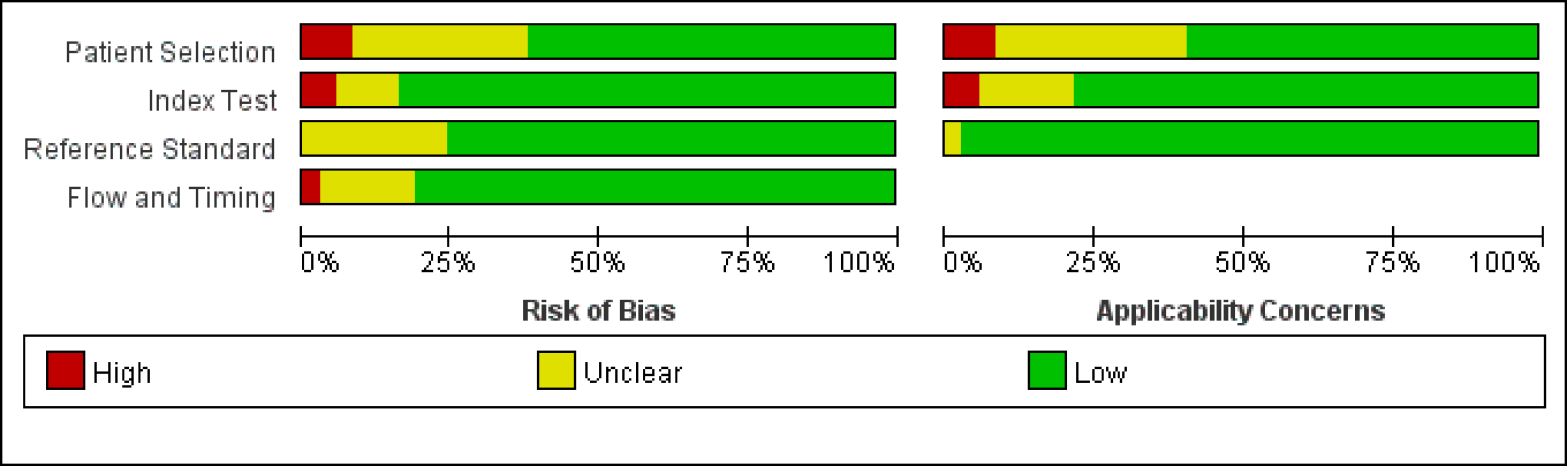
Graphical display for Quality Assessment of Diagnostic Accuracy Studies (QUADAS-2) results regarding the proportion of studies with low, high, or unclear risk of bias.

A preliminary database search yielded 1,970 articles, of which 163 were considered relevant for a full test assessment. After screening and data extraction to evaluate whether the articles were suitable for inclusion, 37 eligible items were included in this meta-analysis (9, 11, 22–56). The research selection flowchart is presented in **Figure 1**. The characteristics of the studies are presented in **Table 1**. The reference standards were divided into the following five categories according to different morphological criteria: (A) a short-axis diameter of 5 mm (22–34), (B) morphological standard, including an irregular border and mixed-signal intensity within the lymph node (35–40), (C) a short-axis diameter of 5 mm with the morphological standard (11, 41–48), (D) a short-axis diameter of 8 mm with the morphological standard (49–52), and (E) a short-axis diameter of 10 mm with the morphological standard (11, 45, 53, 54). In all of the included articles, 36 indirectly evaluated the lymph node stage of patients through histopathology and 5 (9, 41, 42, 55, 56) identified the node-by-node correspondence between lymph node MRI scans and histopathologic results. Across all studies analyzed, 2,875 patients and 983 lymph nodes were included. **Table 2** shows the details of the quality assessment. **Figure 2** gives a graphical display for QUADAS-2 results regarding the distribution of the risk of bias.

**Table 1:** Characteristics of studies included in the analysis.

Study	Year	Country	Design	Assessment approach, No. of readers for each MRI	Field strength	High resolution	Blinding	Reference standard	IC	PN/LN
Xu et al. (22)	2020	China	P	Consensus	3.0	Y	D	H, S	5mm,short-axis	354
Xu HS et al. (49)	2021	China	P	Consensus	3.0	Y	D	H, S	8mm,short-axis+MS	120
Tersteeg et al. (23)	2020	Netherlands	R	Consensus	1.5	N	Y	H, S	5mm,short-axis	324
Iannicelli et al. (24)	2014	Italy	P	Consensus	1.5	Y	D	H, S	5mm,short-axis	73
White et al. (25)	2013	Australia	R	Consensus	1.5	N	Y	H, S	5mm,short-axis	58
Park et al. (41)	2014	Korea	P	Consensus	3.0	Y	D	H, S	5mm,short-axis+MS	40/205LN
Lambregts et al. (42)	2011	Netherlands	R	Consensus	1.5	N	D	H, S	5mm,short-axis+MS	26/111LN
Kim et al. (50)	2011	Korea	R	Independent	3.0	Y	D	H, S	8mm,short-axis+MS	30
Fernández-Esparrach et al. (35)	2011	Spain	P	Consensus	3.0	Y	D	H, S	MS	90
Koh et al. (55)	2010	United Kingdom	P	Consensus	1.5	N	D	H, S	MS	126LN
Jao et al. (36)	2010	Taiwan	P	Consensus	1.5	N	D	H, S	MS	37
Zhang et al. (51)	2007	China	P	Consensus	1.5	N	D	H, S	8mm,short-axis+MS	53
Winter et al. (26)	2007	Germany	P	Consensus	3.0	Y	D	H, S	5mm,short-axis	21
Tatli et al. (27)	2006	USA	R	Independent	1.5	N	D	H, S	5mm,short-axis	25
Song et al. (53)	2018	China	R	Consensus	1.5	N	D	H, S	10mm,short-axis+MS	84
Rafaelsen et al. (28)	2008	Danish	R	Consensus	1.5	N	D	H, S	5mm,short-axis	134
Matsuoka et al. (29)	2003	Japan	P	Independent	1.5	N	D	H, S	5mm,short-axis	21
Kocaman et al. (30)	2014	Turkey	R	Consensus	1.5	N	D	H, S	5mm,short-axis	50
Kim MJ et al. (52)	2008	Korea	R	Consensus	3.0	Y	D	H, S	8mm,short-axis+MS	42
Kim et al. (54)	2000	Korea	R	Independent	1.5	N	Y	H, S	10mm,short-axis+MS	217
Kim JH et al. (11)	2004	Netherlands	P	Independent	1.5	Y	D	H, S	5mm+MS/10mm+MS	75
Kim et al. (56)	2006	Korea	P	Consensus	3.0	N	D	H, S	MS	257LN
Jiang et al. (43)	2006	China	P	Consensus	3.0	Y	D	H, S	5mm,short-axis+MS	53
Halefoglu et al. (31)	2008	Turkey	P	Independent	1.5	N	D	H, S	5mm,short-axis	34
Gagliardi et al. (32)	2002	England	R	Independent	1.5	N	Y	H, S	5mm,short-axis	28
Chun et al. (37)	2006	Korea	P	Consensus	3.0	Y	D	H, S	MS	24
Algebally et al. (44)	2015	Egypt	P	Independent	1.5	Y	Y	H, S	5mm,short-axis+MS	56
Armbruster et al. (45)	2018	Germany	P	Consensus	1.5	N	D	H, S	5mm+MS/10mm+MS	22
Halefoglu et al. (46)	2013	Turkey	P	Independent	1.5	N	D	H, S	5mm,short-axis+MS	93
Kim et al. (38)	2007	Korea	P	Consensus	3.0	Y	D	H, S	MS	26
Bogach et al. (39)	2017	Canada	R	Consensus	3.0	N	Y	H, S	MS	109
Akasu et al. (47)	2009	Japan	P	Consensus	1.5	Y	D	H, S	5mm,short-axis+MS	104
Gröne et al. (33)	2017	Germany	R	Consensus	1.5	Y	D	H, S	5mm,short-axis	60
Brown et al. (9)	2003	England	P	Consensus	1.5	N	D	H, S	5mm,short-axis+MS	284LN
Ferri et al. (34)	2005	Italy	R	Consensus	1.5	N	D	H, S	5mm,short-axis	29
Kim MJ et al. (48)	2004	Korea	P	Independent	1.5	N	D	H, S	5mm,short-axis+MS	62
Kim JH et al. (11)	2009	Korea	P	Independent	1.5	N	D	H, S	MS	66

P, prospective; R, retrospective; Y, yes; N, no; D, double blinding; H, histologic diagnosis; S, surgery; IC, interpretation criteria; MS, morphological standards; PN, patient number; LN, lymph nodes.

**Table 2:** Quality assessment of the 37 included diagnostic studies.

Study Authors	Year	Risk of bias			Flow and timing	Applicability concerns		
		Patient selection	Index test	Reference standard		Patient selection	Index test	Reference standard
Xu et al. (22)	2020	+	+	+	+	+	+	+
Xu HS et al. (49)	2021	+	+	+	?	+	+	+
Tersteeg et al. (23)	2020	?	–	?	?	?	–	+
Iannicelli et al. (24)	2014	+	+	+	+	+	+	+
White et al. (25)	2013	?	+	?	+	?	+	+
Park et al. (41)	2014	?	+	+	+	?	–	+
Lambregts et al. (42)	2011	+	+	+	+	+	+	+
Kim et al. (50)	2011	?	+	+	+	?	+	+
Fernández-Esparrach et al. (35)	2011	+	+	?	–	?	+	?
Koh et al. (55)	2010	+	+	+	+	+	+	+
Jao et al. (36)	2010	+	+	+	+	+	+	+
Zhang et al. (51)	2007	–	+	+	?	–	+	+
Winter et al. (26)	2007	?	+	+	?	?	+	+
Tatli et al. (27)	2006	?	+	+	+	?	+	+
Song et al. (53)	2018	+	+	+	+	+	+	+
Rafaelsen et al. (28)	2008	+	+	?	+	+	+	+
Matsuoka et al. (29)	2003	?	+	+	+	?	+	+
Kocaman et al. (30)	2014	–	+	+	+	–	+	+
Kim et al. (52)	2008	+	+	+	+	+	+	+
Kim et al. (54)	2000	?	+	+	+	?	+	+
Kim JH et al. (11)	2004	?	+	+	+	?	+	+
Kim et al. (56)	2006	+	+	+	+	+	+	+
Jiang et al. (43)	2006	+	+	+	+	+	+	+
Halefoglu et al. (31)	2008	+	+	+	+	+	+	+
Gagliardi et al. (32)	2002	+	?	?	+	+	?	+
Chun et al. (37)	2006	+	+	+	+	+	+	+
Algebally et al. (44)	2015	+	?	?	+	+	?	+
Armbruster et al. (45)	2018	–	?	+	+	–	?	+
Halefoglu et al. (46)	2013	+	+	+	+	+	?	+
Kim et al. (38)	2007	+	+	+	+	+	+	+
Bogach et al. (39)	2017	?	–	?	+	?	?	+
Akasu et al. (47)	2009	+	+	+	+	+	+	+
Gröne et al. (33)	2017	+	+	+	+	+	+	+
Brown et al. (9)	2003	+	+	+	?	+	+	+
Ferri et al. (34)	2005	?	+	+	+	?	+	+
Kim MJ et al. (48)	2004	+	?	?	+	+	?	+
Kim JH et al. (11)	2009	+	+	?	+	+	+	+

+, low risk; -, high risk; ?, unclear risk.

### Diagnostic Performance

**Figure 3:**
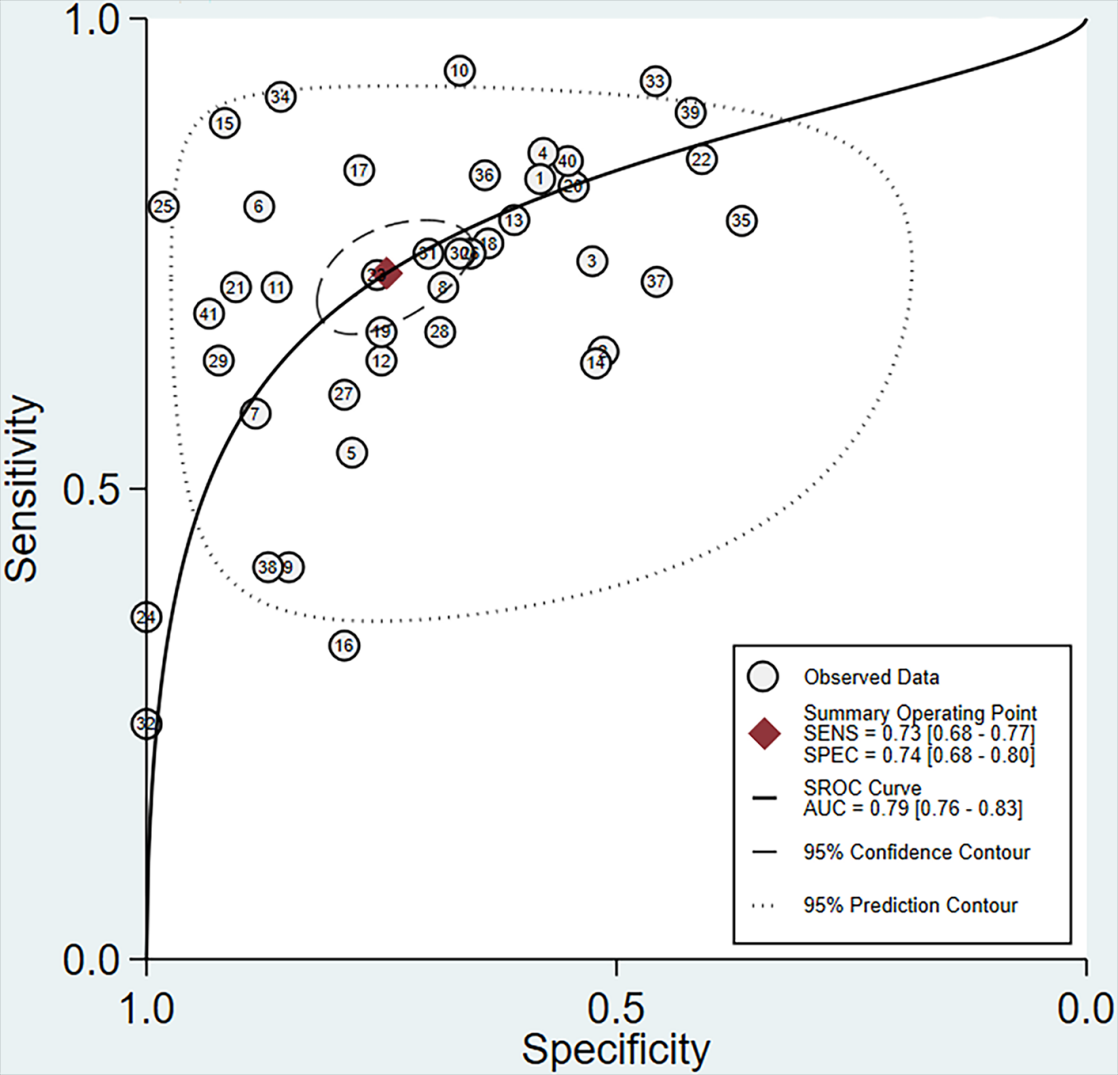
Summary receiver operating characteristic (SROC) curve for MRI assessment of lymph node metastasis in rectal cancer.

The pooled sensitivity and specificity of MRI in the comprehensive diagnosis of metastatic lymph nodes were 0.73 (95% confidence interval [CI], 0.68–0.77) and 0.74 (95% CI, 0.68–0.80), respectively. The pooled sensitivity, specificity, diagnostic odds ratio, positive likelihood ratio, and negative likelihood ratio with corresponding 95% CIs are listed in **Table 3**. The area under the ROC curve (AUC) value of the fitted summary ROC was 0.7877 (**Figure 3**).

**Table 3:** The pooled sensitivity, specificity, PLR, and NLR with corresponding 95% CIs for each included study under different morphological standards.

Index test	SEN (95% CI)	SPE (95% CI)	DOR (95% CI)	PLR (95% CI)	NLR (95% CI)	AUC
Total	0.73 (0.68-0.77)	0.74 (0.68-0.80)	7.85 (5.78-10.66)	2.85 (2.27-3.58)	0.36 (0.31-0.42)	0.79 (0.76-0.83)
5MM	0.75 (0.67-0.81)	0.64 (0.57-0.71)	5.20 (3.76-7.18)	2.07 (1.76-2.43)	0.40 (0.31-0.50)	0.75 (0.71-0.78)
MS	0.74 (0.67-0.80)	0.79 (0.58-0.91)	10.86 (4.19-28.13)	3.57 (1.65-7.74)	0.33 (0.25-0.43)	0.77 (0.73-0.81)
5MM+MS	0.81 (0.74-0.87)	0.67 (0.58-0.74)	8.53 (5.59-13.01)	2.42 (1.94-3.03)	0.28 (0.21-0.39)	0.81 (0.78-0.85)
8MM+MS	0.72 (0.60-0.82)	0.66 (0.47-0.81)	5.18 (1.60-16.80)	2.15 (1.16-3.99)	0.42 (0.23-0.75)	0.76 (0.72-0.79)
10MM+MS	0.62 (0.34-0.83)	0.91 (0.51-0.99)	16.21 (3.74-70.21)	6.80 (1.22-37.81)	0.42 (0.24-0.72)	0.81 (0.77-0.84)

MS, morphological standards; DOR, diagnostic odds ratio; PLR, positive likelihood ratio; NLR, negative likelihood ratio; AUC, area under the curve.

**Figure 4:**
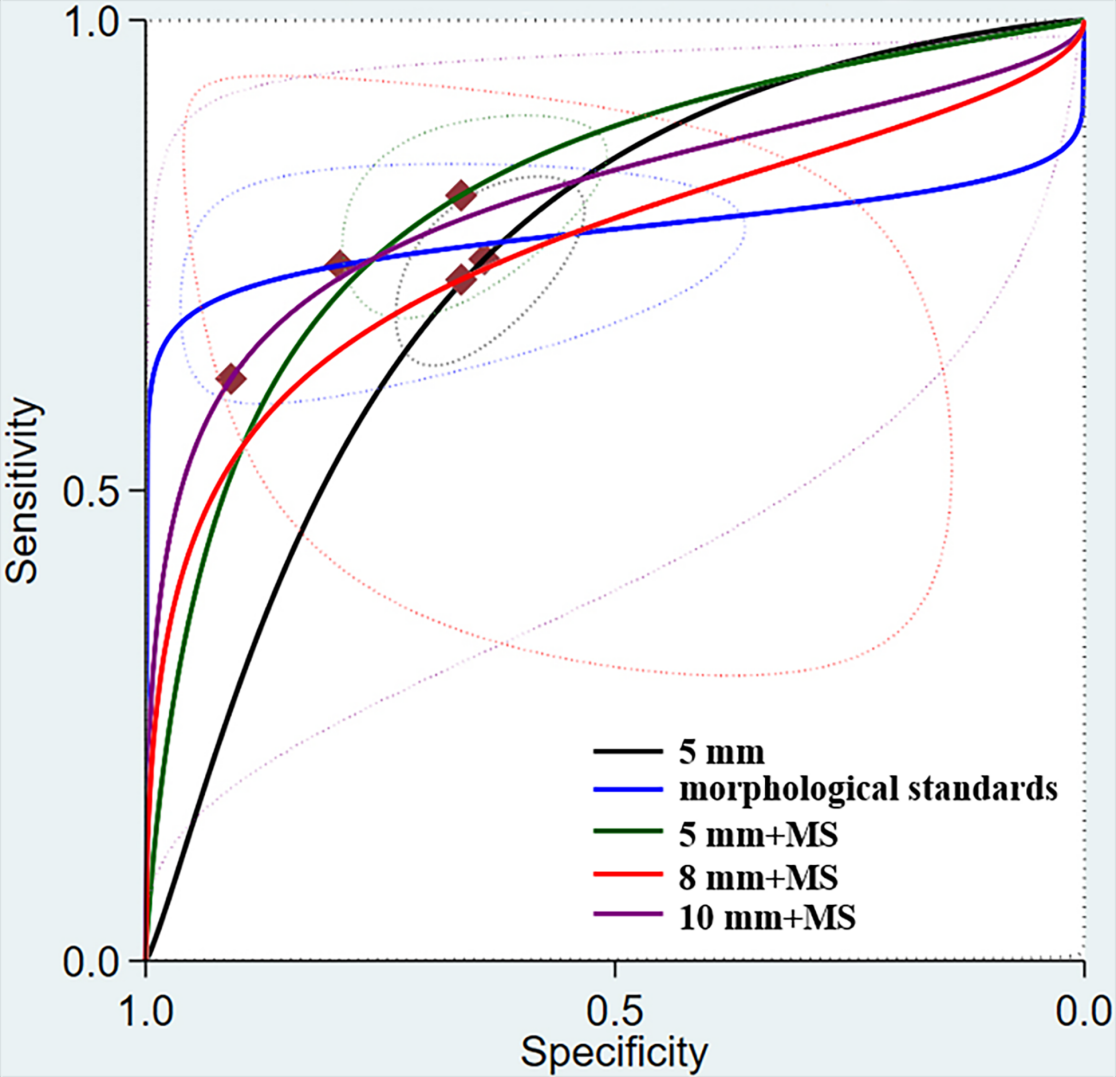
Summary receiver operating characteristic (SROC) curve for MRI assessment of lymph node metastasis under different morphological standards.

Among the different morphological criteria, “a short-axis diameter of 5 mm with the morphological standard” revealed the highest sensitivity of 0.81 (95% CI, 0.74–0.87), and “a short-axis diameter of 10 mm with the morphological standard” revealed the highest specificity of 0.91 (95% CI, 0.51–0.99) (**Table 3**). The AUCs indicated a diagnostic accuracy rate of 0.75–0.81. The morphological standards with the highest accuracy were “a short-axis diameter of 5 mm with the morphological standard” and “a short-axis diameter of 10 mm with the morphological standard” (**Figure 4**).

### Test of Heterogeneity and Metaregression Analysis

The heterogeneity tests showed that the Spearman’s correlation coefficient was 0.446 (p = 0.004), indicating the presence of a threshold effect. This means that different evaluation criteria have led to a significant heterogeneity. Under different morphological standards, there is considerable heterogeneity among 1) the morphological standard, 2) a short-axis diameter of 8 mm with the morphological standard, and 3) a short-axis diameter of 10 mm with the morphological standard (all p < 0.05, i2 > 50%). Therefore, in addition to the threshold effect, there must be other factors that cause significant heterogeneity. A single-factor meta-regression analysis was performed on all the elements. The results showed that the blinding procedure had a particular impact on the heterogeneity of the research (**Table 4**).

**Table 4:** Results of subgroup analysis for evaluation of all studies.

**Study characteristics**	**No.**	**Pooled sensitivity** **(95% CI)**	**Pooled specificity (95% CI)**	**Positive LR (95% CI)**	**Negative LR (95% CI)**	**AUC**	**p**
Total	41	0.73 (0.68-0.77)	0.74 (0.68-0.80)	2.85 (2.27-3.58)	0.36 (0.31-0.42)	0.7877	
Field strength, Tesla							
1.5	28	0.72 (0.69-0.75)	0.70 (0.67-0.72)	2.04 (1.78-2.33)	0.46 (0.39-0.55)	0.7559	0.0524
3.0	13	0.77 (0.73-0.80)	0.78 (0.75-0.81)	3.92 (2.49-6.18)	0.35 (0.27-0.46)	0.8412	
High resolution							
Yes	17	0.74 (0.67-0.80)	0.78 (0.67-0.86)	3.30 (2.27-4.80)	0.34 (0.28-0.41)	0.8125	0.2513
No/Not specified	24	0.72 (0.65-0.81)	0.73 (0.64-0.81)	2.73 (2.03-3.66)	0.37 (0.30-0.47)	0.7936	
Design							
Retrospective	15	0.77 (0.73-0.81)	0.62 (0.58-0.66)	1.85 (1.53-2.22)	0.46 (0.37-0.58)	0.7421	0.1358
Prospective	26	0.72 (0.69-0.75)	0.77 (0.75-0.79)	2.87 (2.28-3.62)	0.40 (0.33-0.49)	0.8056	
Node by node							
Yes	5	0.55 (0.40-0.69)	0.89 (0.79-0.95)	5.21 (2.03-13.46)	0.51 (0.34-0.76)	0.7813	0.9405
No	36	0.74 (0.70-0.79)	0.71 (0.64-0.77)	2.59 (2.12-3.10)	0.36 (0.31-0.42)	0.7937	
Read approach							
Independent	12	0.77 (0.72-0.81)	0.64 (0.60-0.69)	2.14 (1.65-2.77)	0.42 (0.31-0.55)	0.7853	0.6774
Consensus	29	0.73 (0.70-0.76)	0.75 (0.73-0.77)	2.53 (2.06-3.10)	0.42 (0.35-0.51)	0.7894	
Blinding							
Single	7	0.72 (0.63-0.80)	0.57 (0.46-0.67)	1.70 (1.40-2.03)	0.49 (0.38-0.63)	0.7008	0.0281
Double	34	0.73 (0.67-0.78)	0.78 (0.70-0.84)	3.31 (2.54-4.28)	0.34 (0.29-0.41)	0.8082	

No., number of data subsets; AUC, area under the curve; p, p value of meta-regression analysis.

### Subgroup Analysis

Subgroup analyses were performed for the different study characteristics. By comparing references with or without node-by-node correspondence, we found that a lower sensitivity of 0.55 (95% CI, 0.40–0.69) and higher specificity of 0.89 (95% CI, 0.79–0.95) were yielded. When considering different MRI types, both 3.0T and high-resolution MRI yielded a higher sensitivity and specificity. Through a subgroup analysis of the study design, read approach, and blinding procedure, studies that used double blinding yielded a higher sensitivity of 73% (95% CI, 0.67–0.78) and specificity of 78% (95% CI, 0.70–0.84), whereas prospective studies yielded a higher specificity of 77% (95% CI, 0.75–0.79). The results of the subgroup analysis are shown in **Table 4**.

### Sensitivity Analysis

**Figure 5:**
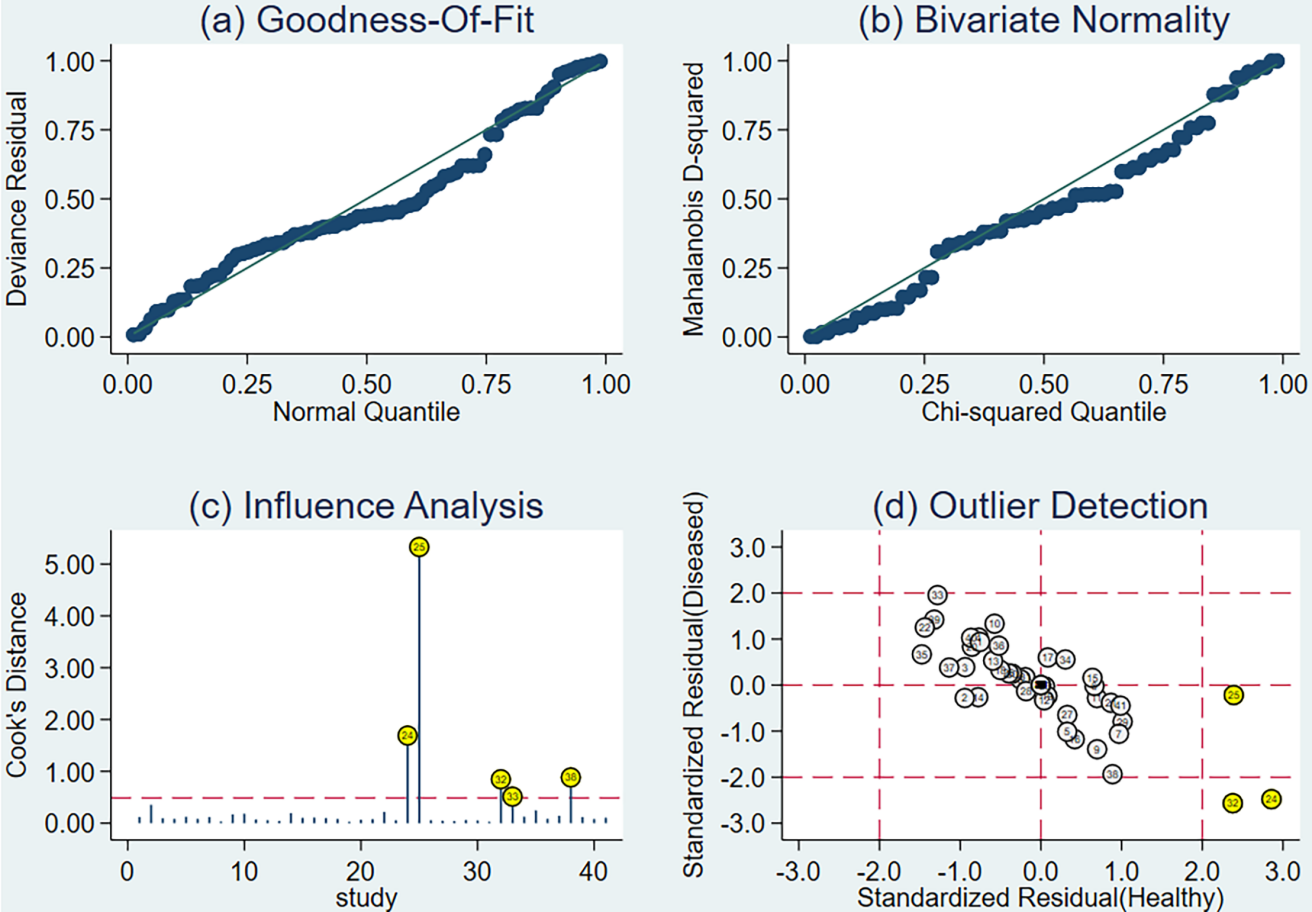
Sensitivity analysis results of all studies: **(A)** goodness of fit, **(B)** bivariate normality, **(C)** influence analysis, and **(D)** outlier detection.

A sensitivity analysis of all the studies revealed that five original studies had a strong sensitivity (**Figure 5**), whereas the other original studies did not strongly affect the calculation results. After excluding the literature mentioned above, the other 36 sub-datasets still had threshold effects. The pooled sensitivity, specificity, positive likelihood ratio, negative likelihood ratio, and diagnostic odds ratio were 0.74 (95% CI, 0.71–0.78), 0.70 (95% CI, 0.64–0.75), 2.45 (95% CI, 2.08–2.89), 0.37 (95% CI, 0.32–0.42), and 6.67 (95% CI, 5.23–8.48), respectively. Further, the AUC was 0.7750.

### Publication Bias

**Figure 6:**
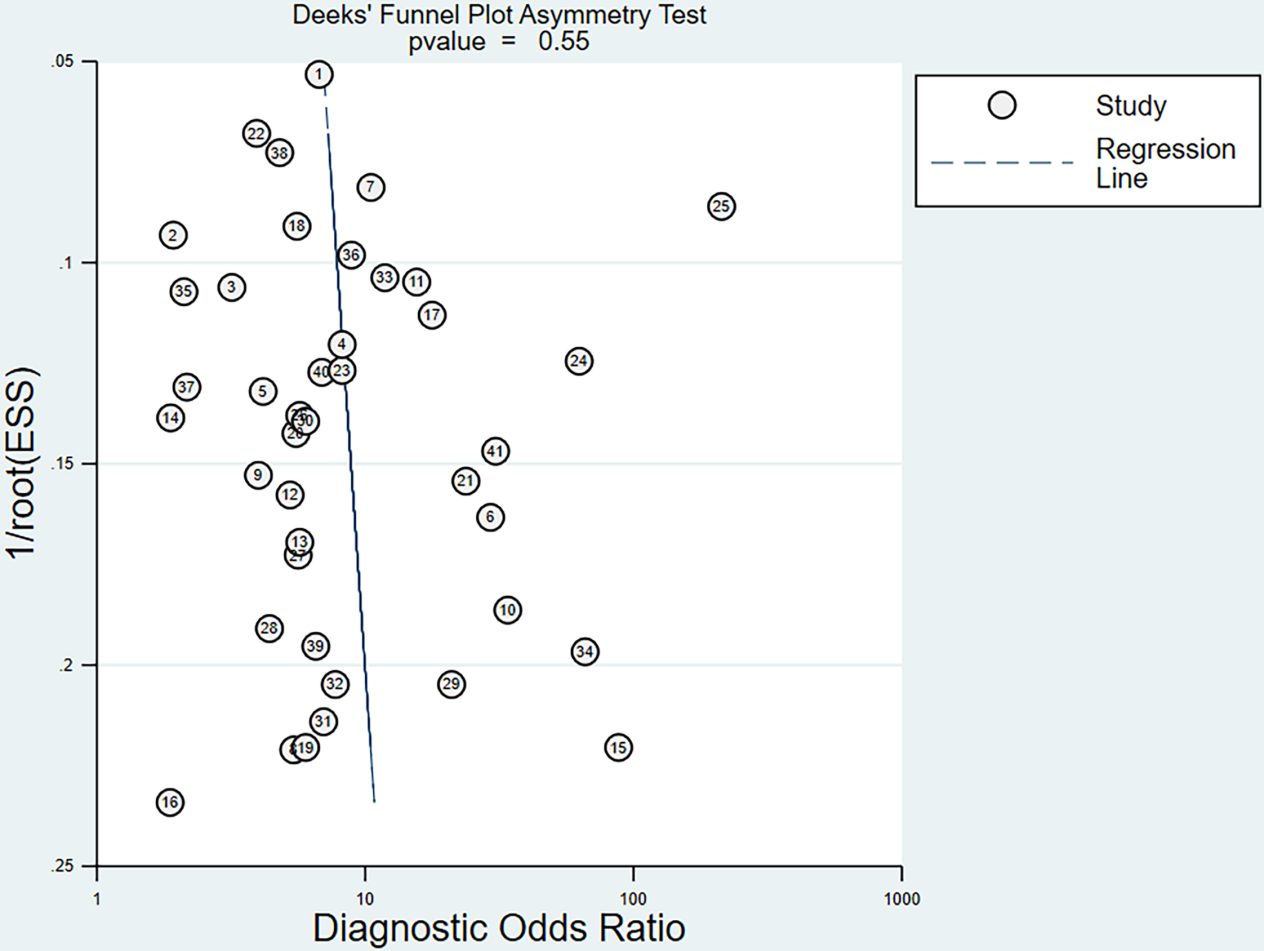
Funnel plot of the reciprocal of effective sample size (ESS) plotted on the y-axis against the diagnostic odds ratio plotted on the x-axis. The regression line is used as a measure of asymmetry. The circles represent included studies.

For all studies, the p-value of the bias on the Deek’s funnel plot asymmetry test was 0.55, indicating that these studies did not have significant publication bias (**Figure 6**).

## Discussion

Lymph node status plays a vital role in selecting treatment strategies for colorectal cancer, with the presence or absence of regional lymph node metastasis being the key to treatment selection. The advantage of MRI is that it can identify the mesorectal fascia, enabling accurate preoperative identification of patients with lymph nodes that cannot be entirely surgically removed. Therefore, in the context of neoadjuvant therapy, preoperative MRI must provide an accurate diagnosis of regional lymph nodes, avoid overestimation and underestimation before treatment, and provide the optimal treatment decision for individual patients. In this study, we evaluated the ability of MRI to determine the lymph node stage of rectal cancer. The results showed that the value of MRI in diagnosing metastatic lymph nodes was low (57–59).

These findings are similar to those reported by Al-Sukhni et al. (15–17), who concluded that MRI only moderates the diagnostic ability for lymph node metastasis. It is worth noting that the previous meta-analysis found significant heterogeneity in the assessment of lymph node metastasis and speculated that the threshold effect is the primary source of heterogeneity. Therefore, we corrected for some of the limitations recognized by previous studies by including more original articles and classifying lymph nodes for statistical analysis based on different morphological standards.

Most MRI studies on colorectal cancer published have used lymph node size as a standard criterion for predicting lymph node involvement. However, previous studies demonstrated that using only the size of lymph nodes as a criterion does not improve the accuracy of lymph node staging of colorectal cancer (9–11), which is consistent with our results. We found that there was no significant difference in the accuracy of MRI diagnosis when using different standards. It is worth mentioning that under the same morphological standard, as the shorter diameter of the lymph node increases, the sensitivity gradually decreases and the specificity gradually increases (**Table 3**). This may be because although malignant lymph nodes usually have a larger short-axis diameter than benign lymph nodes, there is a considerable size overlap between benign and malignant lymph nodes, with approximately 30% of metastatic lymph nodes having a diameter of ≤4 mm (12). In addition, benign lymph nodes may appear to increase in size with the development of fibrosis (60).

Compared with the size standard alone, different morphological features have been previously considered as good criteria for judging metastatic lymph nodes. Brown et al. first described the use of MRI to improve the correct diagnosis of lymph node involvement in rectal cancer when boundary contours and signal intensity features were used instead of size standards alone (9). Kim et al. demonstrated that in addition to size, new criteria, such as burr-like or inconspicuous borders and uneven appearance, can be used to predict regional lymph node involvement (11). Their results were better than our findings. We found that after adding morphological features, the pooled sensitivity and specificity of lymph node diagnosis improved. However, the diagnostic performance did not improve significantly (**Table 3**), possibly because the morphological characteristics are more subjective among different observers.

We found that both high-field strength (3.0 Tesla) and high-resolution MRI yielded a higher sensitivity and specificity than low-field strength (1.5 Tesla) according to a subgroup analysis (**Table 4**). Due to the retrospective design of the research, patient selection, and MRI plan, the diagnostic performance of prospectively designed research was slightly better in the subgroup analysis. In addition, double-blind studies had a higher specificity than single-blind studies (0.78, 95% CI 0.70–0.84). As with other diagnostic meta-analyses, heterogeneity is a vital limitation among studies, including study design, MRI protocols, blinding procedures, and reference standards. In the regression analysis, we found that the blinding procedure (single-blind/double-blind) helps assess heterogeneity, leading to differences among research conclusions.

In most previous studies, the assessment of the lymph node staging of patients mainly relied on the number of positive lymph nodes found in the mesorectum after the overall sampling of rectal specimens, which does not have a high accuracy and reliability. Thus far, few studies have reported that the individual lymph nodes seen on MRI scans match the exact pathological correspondence after rectal resection. We included five references as subgroups. Our analysis found that the sensitivity of MRI for the diagnosis of a single lymph node decreased, the specificity significantly improved, and the accuracy of the assessment was lower than expected. The possible reasons for the inconsistent diagnostic accuracy could be due to small number of references, a lack of consistency in the threshold, and the difference in the realization of node-by-node correspondence.

Currently, new technologies are being explored to improve preoperative staging. The chemical shift effect is a reliable indicator for identifying benign and malignant lymph nodes (61), and Farshchian first proposed that it has the potential to diagnose benign lymph nodes (62). Grovik et al. showed that a low K^trans^ of the primary tumor can predict the presence of nodal metastasis (63), which can be achieved by dynamic contrast-enhanced MRI (DCE-MRI) (64, 65). In addition to DCE-MRI, special diffusion-weighted MRI parameters are helpful in differentiating metastatic lymph nodes (45, 66).

The use of lymphatic contrast agents is considered a method for improving the staging of lymph nodes. USPIO is the most widely used contrast agent (67, 68). This technology allows for the differentiation of malignant and benign lymph nodes according to the contrast-enhanced pattern. Although MRI with USPIO has achieved some success in characterizing small lymph nodes, further research is needed regarding its clinical applicability (55, 69–71).

Radiomics is a rapidly developing discipline that uses computer algorithms to extract quantitative features from MRI scans (72–74). These algorithms capture the image texture and morphology of tumors based on their gray values. Since 2018, many reports on radiological methods for rectal cancer lymph node assessment have been published (75–78). However, when analyzing imaging information and building predictive models, all these parameters require time-consuming calculations. In the future, artificial intelligence is expected to become the optimal option for determining lymph node staging and treatments options for patients with locally advanced rectal cancer.

Recently, the importance of lymph node metastasis in the process of tumor recurrence has begun to be questioned, i.e., the indications of neoadjuvant therapy are not based on clinical TNM staging. Additionally, determining whether there are other prognostic markers detected by MRI, such as extra-mural venous invasion (EMVI) and circumferential resection margin (CRM), is more important (79–81). The MERCURY study showed that lymph node involvement is not an independent predictor of local recurrence, and using CRM was recommended for evaluating neoadjuvant therapy (82). In this case, clinical lymph node assessment for rectal cancer may only play a secondary role in guiding future treatment decisions (83, 84).

This study has some limitations. First, our meta-analysis included 37 studies and 2,875 patients. Although this is a comprehensive literature search, more studies may provide more accurate estimates and comparisons of results. Second, the content of some reports is insufficient, limiting our quality assessment and individual analysis of more subgroups. Finally, heterogeneity is still an essential issue in meta-analyses. In future studies, the definition of critical staging elements and MRI protocols should be standardized to reduce heterogeneity. Therefore, considering the limitations of diagnostic meta-analysis, the results should be interpreted prudently.

## Conclusion

In summary, the performance of MRI in the detection of lymph node metastasis is inadequate, and either through using more morphological characteristics or shorter diameter, is not significantly improved. At present, when making preoperative neoadjuvant treatment decisions, evidence from a variety of imaging methods should be combined to determine the optimal treatment strategy.

## Data Availability Statement

The original contributions presented in the study are included in the article/supplementary material. Further inquiries can be directed to the corresponding author.

## Author Contributions

ZZ contributed the most to this article. ZZ designed the project, developed the search strategy and wrote the manuscript. ZZ and YZ checked the search, and reviewed the manuscript. MW performed literature screening and data extraction, conduct the quality assessment of the included studies. XY carried out the data analysis. ZW reviewed the manuscript and finally approved the version to be published. All authors contributed to the article and approved the submitted version.

## Funding

The work is supported by the Department of Science and Technology of Sichuan Province (2019YFS0375; 2018RZ0091; 2018SZ0242; 2021YFS0025)

## Conflict of Interest

The authors declare that the research was conducted in the absence of any commercial or financial relationships that could be construed as a potential conflict of interest.

## Abbreviations

MRI, magnetic resonance imaging; RC, rectal cancer; LN, lymph nodes; CRM, circumferential resection margin; EMVI, extramural venous invasion; AJCC, American Joint Commission on Cancer; NCCN, National Comprehensive Cancer Network; QUADAS-2, Quality assessment of Diagnostic accuracy studies-2; CI, confidence interval; AUC, area under the ROC curve; SROC, summary receiver operating characteristics; USPIO, ultrasmall particles of iron oxide; DCE-MRI, dynamic contrast-enhanced MRI.
